# Dehydrocostus Lactone Attenuates the Senescence of Nucleus Pulposus Cells and Ameliorates Intervertebral Disc Degeneration via Inhibition of STING-TBK1/NF-κB and MAPK Signaling

**DOI:** 10.3389/fphar.2021.641098

**Published:** 2021-04-14

**Authors:** Zhiqian Chen, Xiao Yang, Yifan Zhou, Zhihao Liang, Chen Chen, Chen Han, Xiankun Cao, Wenxin He, Kai Zhang, An Qin, Tangjun Zhou, Jie Zhao

**Affiliations:** ^1^ Shanghai Key Laboratory of Orthopaedic Implant, Shanghai Ninth People’s Hospital, Shanghai, China; ^2^ Department of Orthopedics, Ninth People’s Hospital, Shanghai Jiaotong University School of Medicine, Shanghai, China

**Keywords:** intervertebral disc degeneration, inflammation, dehydrocostus lactone, senescence, nucleus pulposus cells

## Abstract

The progression of intervertebral disc degeneration (IDD) is multifactorial with the senescence of nucleus pulposus (NP) cells and closely related to inflammation in NP cells. Dehydrocostus lactone (DHE) is a natural sesquiterpene lactone isolated from medicinal plants that has anti-inflammatory properties. Thus, DHE may have a therapeutic effect on the progression of IDD. In this study, NP cells were used to determine the appropriate concentration of DHE *in vitro*. The role of DHE in tumor necrosis factor-α (TNF-α)–induced activation of inflammatory signaling pathways and cellular senescence, together with anabolism and catabolism of extracellular matrix (ECM) in NP cells, was examined *in vitro*. The therapeutic effect of DHE *in vivo* was determined using a spinal instability model of IDD in mice. The TNF-α–induced ECM degradation and the senescence of NP cells were partially attenuated by DHE. Mechanistically, DHE inhibited the activation of NF-κB and MAPK inflammatory signaling pathways and ameliorated the senescence of NP cells caused by the activation of STING-TBK1/NF-κB signaling induced by TNF-α. Furthermore, a spinal instability model in mice demonstrated that DHE treatment could ameliorate progression of IDD. Together, our findings indicate that DHE can alleviate IDD changes and has a potential therapeutic function for the treatment of IDD.

## Introduction

Lumbar intervertebral disc degeneration (IDD) is the major underlying cause of low back pain (D'Silva et al., 2019) and usually precedes other spinal degenerative disorders, including lumbar disc herniation and spinal stenosis, which result in a significant economic burden and enormous influences on quality of life (Safiri et al., 2020). The occurrence and development of IDD are derived from aging, acute trauma, genetic predisposition, smoking, inflammation, abnormal biomechanical loading, decreased nutrient transport across the endplate, and hyperglycemia (Alpantaki et al., 2019). The intervertebral disc (IVD) is composed of a gel-like nucleus pulposus (NP) tissue in the center, and the NP cells play a core role for the secretion of extracellular matrix (ECM) proteins, which mainly include aggrecan and type II collagen. The ECM has the key functions of maintaining the disc height and relieving the axial mechanical pressure of the body (Roberts et al., 2006).

NP cell senescence, inflammation, and ECM degradation are considered the main molecular mechanisms of IDD (Feng et al., 2016; Zhang et al., 2016). Cell senescence and inflammatory response lead to the catabolic upregulation of NP cells, which results in the imbalance of ECM metabolism and, finally, causes ECM degradation. Tumor necrosis factor-α (TNF-α) is the key inflammatory factor that accelerates senescence of NP cells and induces the expression of other inflammatory mediators (Postal and Appenzeller, 2011; Johnson et al., 2015; Khan et al., 2017); as a result, matrix metalloproteinase (MMP) increases and, ultimately, loss of ECM occurs and IDD develops (Sun et al., 2015). Therefore, effective inhibition of TNF-α, of TNF-α–induced NP cell senescence, and of inflammatory mediators may treat IDD.

TNF-α induces the upregulation of inflammatory cytokines and causes DNA damage (Fehaid and Taniguchi, 2019; Kay et al., 2019; Shao et al., 2019). These changes result in double-stranded DNA (dsDNA) increases in the cytoplasm of cells, which comprise a phenotype—the senescence-associated secretory phenotype (SASP)—that involves cell senescence and abnormal inflammatory factor infiltration (Choubey and Panchanathan, 2016; Guo et al., 2019). Nuclear factor-kappa B (NF-κB) and mitogen-activated protein kinase (MAPK) pathways are considered the main inflammatory responses to TNF-α stimulation in NP cells (Wang G. et al., 2018). In addition, the stimulator of interferon genes (STING)-TBK1/NF-κB pathway has been identified as the main regulator of inflammatory cytokines when dsDNA is detected, in a situation closely related to cellular senescence (Hopfner and Hornung, 2020; Loo et al., 2020). These signaling pathways create a vicious cycle of inflammatory cascades that accelerate IDD. In recent studies, inhibition of these pathways has effectively treated IDD (Khan et al., 2017; Tao et al., 2020).

Dehydrocostus lactone (DHE) is a natural sesquiterpene lactone isolated from medicinal plants that has antioxidant, anti-inflammatory, antiulcer, and anti-hepatotoxic abilities. Previous studies have reported that DHE significantly suppressed lipopolysaccharide-induced acute lung injury and macrophage activation and suppressed estrogen deficiency–induced osteoporosis through NF-κB and MAPK signaling pathways (Hu et al., 2019; Nie et al., 2019). In this study, we conducted an experiment based on TNF-α–induced inflammation in NP cells and investigated the anti-inflammatory and anti-senescence effects of DHE. We also investigated the underlying mechanism of these effects. Moreover, our *in vivo* experiments of spinal instability in a mouse model confirm the therapeutic effectiveness of DHE for IDD.

## Materials and Methods

### Chemicals and Reagents

For *in vitro* and *in vivo* research, dehydrocostus lactone (purity ≥98.8%) was purchased from Selleck Chemicals (Houston, TX, United States). It was dissolved in DMSO as an 80 mM stock solution and stored at −20°C. To reduce cytotoxicity, the final concentrations of DMSO were less than 0.1%.

### NP Cell Lines

The NP cell lines of Sprague Dawley (SD) rats were provided by Dr. Chen Di from the Department of Orthopedic Surgery, Rush University Medical Center (Chicago, IL, United States). NP cells were cultured in Dulbecco’s modified Eagle medium (DMEM) with 10% fetal bovine serum (FBS) and 1% penicillin-streptomycin (Gibco, Thermo Fisher Scientific, Waltham, MA, United States).

### NP Primary Cells Isolation

After euthanasia via intraperitoneal injection of pentobarbital sodium (50 mg/kg of body weight), NP tissues were obtained from the caudal discs (Co1–Co6) of SD rats (4 weeks old). Those tissues were transferred to a CO_2_ incubator with 0.25% type II collagenase for a 2 h digestion. Then, the cells were resuspended and seeded in a culture plate with DMEM containing 10% FBS and 1% penicillin-streptomycin (Gibco, Thermo Fisher Scientific, Waltham, MA, United States) after centrifugation. All the cells were incubated at 37°C, 21% O_2_, and 5% CO_2_.

### Cell Counting Kit-8 (CCK-8) Assay

Cell toxicity and proliferation were measured using the CCK-8 kit (Sangon Biotech Co. Ltd., Shanghai, China). To determine toxicity, NP cells seeded in 96-well plates at a density of 8 × 10^3^ cells per well were cultured for 24 h with DHE at concentrations of 0, 2.5, 5, 10, 20, or 40 μM. To determine proliferation, NP cells seeded in 96-well plates at a density of 2 × 10^3^ cells per well were cultured for 8, 24, 48, and 72 h. At 8 h, optical density values were recorded on a spectrophotometer, with 450 nm set as the baseline. Then, DHE concentrations of 0, 2.5, 5, 10, 20, or 40 μM were added into the plates. At the end of each experimental period, cells were incubated with 10 μL of the CCK-8 reagent for 1 h at 37°C. Optical density values were recorded on a spectrophotometer at 450 nm on an Infinite M200 Pro multimode microplate reader (Tecan Life Sciences, Männedorf, Switzerland).

### Senescence Assays

Senescent NP primary cells were identified using the senescence *β*-galactosidase staining kit (Cell Signaling Technology, Danvers, MA, United States) in accordance with the manufacturer’s instructions. Briefly, NP cells were treated with 20 ng/ml of TNF-α, with or without DHE (2.5 μM) for 3 days. After 3 days of culture, cells were fixed with the 1X fixative solution for 15 min at room temperature (RT) and then incubated with *β*-galactosidase staining solution at 37°C overnight in a dry incubator without CO_2_. Images were captured under a bright field microscope (Olympus CKX41, JAN). The SA-*β*-Gal–positive cells were measured using Image J software (National Institutes of Health, United States).

### Protein Extraction and Western Blot Analyses

Total cellular proteins were extracted from cultured cells using a RIPA lysis buffer supplemented with phosphatase and protease inhibitors (Roche, Basel, Switzerland). Protein concentrations were quantified using the bicinchoninic acid protein quantification kit (Thermo Fisher Scientific, Waltham, MA, United States). An equal amount of proteins (approximately 25 *μ*g) was resolved on 4–20% SDS-PAGE gel for separation and was electroblotted onto 0.22-*μ*m PVDF membranes (Merck-Millipore, CA, United States). Membranes were blocked with 5% bovine serum albumin–TBST at RT for 1 h and then incubated with primary antibodies (diluted 1:1,000 in 5% BSA–TBST) overnight at 4°C. Primary antibodies included IKKα (D3W6N), IκBα (L35A5), NF-κB p65 (D14E12), SAPK/c-Jun N-terminal kinase (JNK), p38 MAPK (D13E1), STING (D1V5L), phospho-IκBα (Ser32; 14D4) phospho-NF-κB p65 (Ser536), phospho-SAPK/JNK (Thr183/Tyr185; 81E11), phospho-p44/42 MAPK (ERK1/2; Thr202/Tyr204; D13.14.4E), p44/42 MAPK (ERK1/2; 137F5), phospho-IKKα/β (Ser176/180), phospho-p38 MAPK (Thr180/Tyr182; D3F9), and *β*-actin (D6A8). All were purchased from Cell Signaling Technology (Danvers, MA, USA). Additional primary antibodies included anti-NAK/TBK1(ab40676), anti-NAK/TBK1 (phospho S172; ab109272), anti-p21 (ab109199), and anti-p53 (ab26), which were obtained from Abcam (Cambridge, United Kingdom). Membranes were washed extensively in TBST and subsequently incubated with anti-rabbit or anti-mouse IgG (H + L; DyLight™ 800 4× PEG conjugate; Cell Signaling Technology, Danvers, MA, United States) secondary antibody (1:5,000 dilution) for 1 h at RT in the dark. Membranes were washed three times with TBST, and protein immunoreactivity was detected on a LI-COR Odyssey fluorescence imaging system (LI-COR Biosciences, Lincoln, NE, United States). The OD values of each protein were measured using Image J software (National Institutes of Health, United States).

### RNA Extraction and Real-Time Quantitative PCR (RT-qPCR) Analyses

Total RNA was isolated from NP cells using a TRIzol reagent (Thermo Fisher Scientific, Waltham, MA, United States) in accordance with the manufacturer’s protocol. The cDNA was reversed transcribed from extracted RNA using the cDNA synthesis kit (TaKaRa Bio, Otsu, Japan). Real-time qPCR was conducted using the TB Green Premix Ex Taq kit (TaKaRa Bio, Otsu, Japan) on an Applied Biosystems QuantStudio 6 Flex real-time PCR system (Thermo Fisher Scientific, Waltham, MA, United States). Specific primer pairs were designed using NCBI BLAST; sequences are provided in Supplementary Table 1. Target gene expression levels were determined using the 2^−ΔΔCT^ method. The expression of the genes listed in Supplementary Table 1 was normalized to the expression of *β*-actin.

### High-Density Culture

Approximately 150,000 NP primary cells were resuspended in 10 μl of control medium and seeded as micromasses in the bottom of a 12-well plate to assess the ability of ECM secretion of NP cells. NP primary cells were allowed to attach to the bottom for 1 h at 37°C; then, 1 ml of MEM/F12 medium containing 10 ng/ml of ITS (Insulin Transferrin Selenium) and 2% FBS was added. The medium was refreshed every other day; after 9 days, these micromasses were stained with Alcian blue.

### Histology and Immunohistochemistry

Fixed IVD tissue samples were embedded into paraffin blocks and then subjected to histological sectioning (5 μm thickness). For histological assessment, paraffin tissue sections were processed for safranin O-fast green and hematoxylin and eosin staining in accordance with standard laboratory protocols.

For the assessment of immunohistochemistry, tissue sections were deparaffinized in graded xylene and a standard alcohol gradient and then were washed in phosphate-buffered saline (PBS) and water. Then, 10% goat serum was used to block nonspecific binding sites for 30 min at RT. The sections were incubated with the primary antibodies, anti–TNF-α, anti–interleukin (IL)-1β, and anti-aggrecan (purchased from Cell Signaling Technology, Danvers, MA, United States, and Abcam, Cambridge, United Kingdom) overnight at 4°C. The next day, sections were washed with PBS and incubated with an appropriate horseradish peroxidase–labeled secondary antibody (Santa Cruz Biotechnology, Dallas, TX, United States) for 1 h at RT and further developed with diaminobenzidine solution. Subsequently, all sections were captured with a bright field optical microscope. The integrated optical density (IOD) measurements were carried out using Image Pro Plus 6.0 software.

### Immunofluorescence

For immunofluorescence, NP cells were seeded on slides in a six-well plate at a density of 4×10^5^ per well for 8 h. After 8 h, the NP cells were pretreated with or without DHE (2.5 μM) for 2 h and then stimulated with TNF-α (20 ng/ml) for 20 min. For dsDNA in the cytoplasm, the NP cells were treated with TNF-α (20 ng/ml) with or without DHE (2.5 μM) for 8 h. Para-formaldehyde (4%) was used to fix the NP cells for 15 min at RT, and 0.25% Triton X-100 was used to permeabilize the cells membrane for 5 min after washing three times with PBS. After blocking with 10% goat serum, the cells were incubated with a primary antibody for phospho-p65 (diluted 1:200, purchased from Cell Signaling Technology, anvers, MA, USA) or incubated with a primary antibody for dsDNA (diluted 1:200, purchased from Abcam, Cambridge, United Kingdom) overnight at 4°C. The next day, the cells were washed three times with PBS. Then, they were incubated with an Alexa Fluor 594 conjugate secondary antibody (anti-rabbit, 1:500; Cell Signaling Technology, anvers, MA, United States) for 1 h and subsequently incubated with DAPI (Sigma-Aldrich, St. Louis, MO, United States) for 3 min. Finally, digital fluorescence images were captured with a fluorescence microscope (Olympus, Inc., Tokyo, Japan).

### Animals and Surgical Procedures

For the model of lumbar instability, the back hair of the mice was removed with depilating cream. Then, the skin of the back was disinfected and the paraspinal muscles were exposed with a scalpel. The paravertebral muscles on both sides were removed with ophthalmic scissors to expose the spinous process. The spinous processes of L1–L4 were resected with ophthalmic scissors from 12 week-old wile-type (WT) mice on a C57BL/6 background; after careful disinfection and hemostasis, the operative fields were closed with nylon sutures. All surgical procedures were performed under general anesthesia using 3% isoflurane, were approved by the Institutional Animal Care and Ethics Committee of Ninth People’s Hospital, Shanghai Jiaotong University School of Medicine (Shanghai, China), and were performed in accordance with the principles and procedures of the National Institutes of Health Guide for the Care and Use of Laboratory Animals and the Guidelines for Animal Treatment of Shanghai Jiaotong University. After the model was established, the mice were given DHE (20 mg/kg, 120 µl), according to previous research (Wang J. et al., 2017; Hu et al., 2019), or PBS (120 µl) once a day through intraperitoneal injection. Eight weeks after surgery, mice were sacrificed by surgical dislocation, and the lumbar spinal tissues were harvested and fixed in 4% PFA for subsequent downstream analyses. All mice were maintained under pathogen-free conditions.

### Radiographic Analysis

Digital X-ray imaging of the lumbar vertebrae of mice was conducted in the anteroposterior axis with a 21-lp/mm detector that provides up to 5× geometric magnification (Faxitron VersaVision; Faxitron Bioptics, Tucson, AZ, United States). Disc height indexes (DHIs) were calculated according to the following formula: DHI = IVD height/adjacent IVD body height.

### Statistical Analysis

Three independent experiments were performed to obtain all data. The data are presented as the mean ± standard error of the mean. Significance differences between study groups were determined using GraphPad Prism version 7.0 software (GraphPad Software, San Diego, CA, United States). One-way analysis of variance was used to compare the differences between groups. Statistical significance was set at *p* < 0.05.

## Results

### Effects of DHE on Cytotoxicity and Proliferation of NP Cells

**FIGURE 1:**
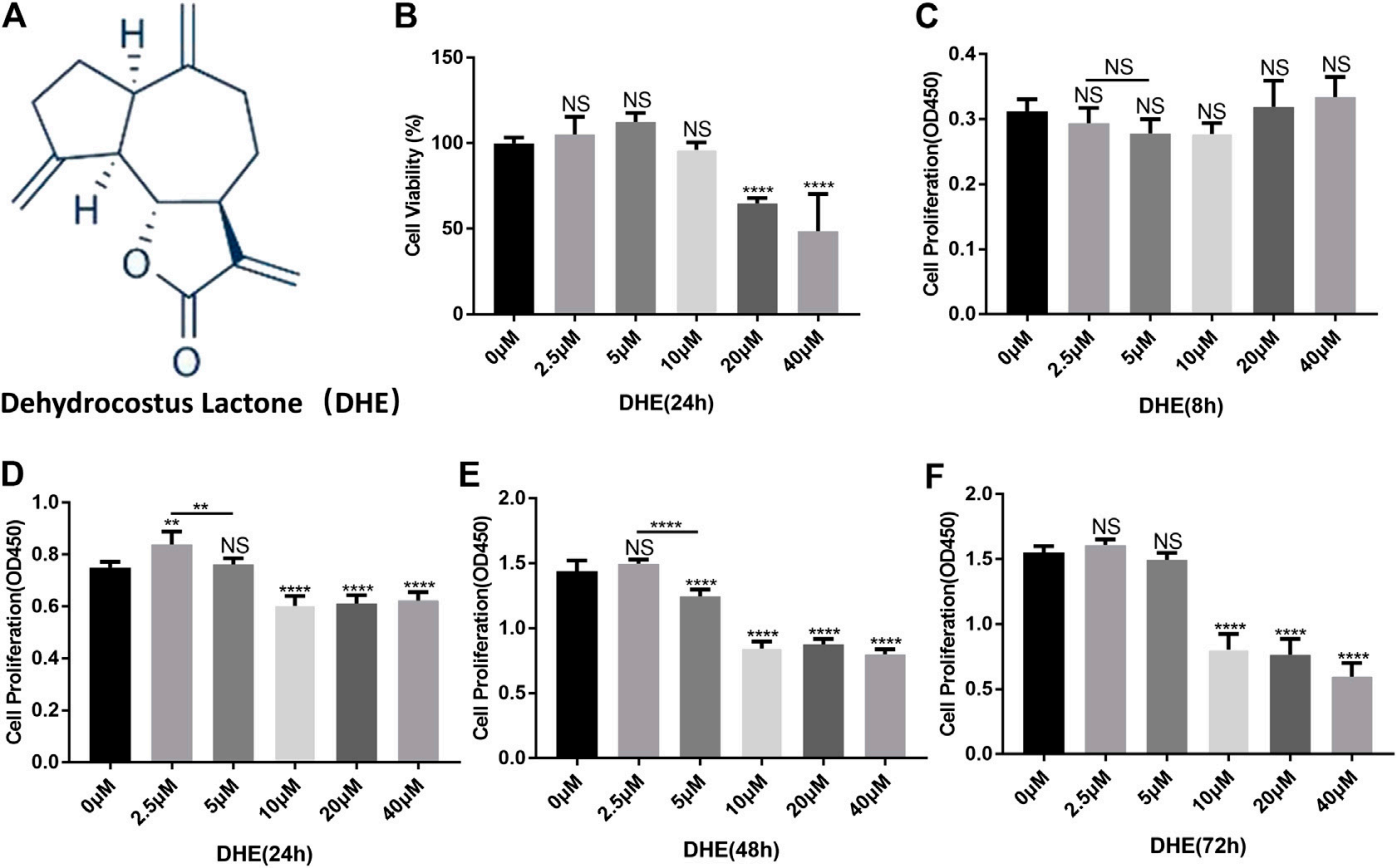
Effects of dehydrocostus lactone (DHE) on the cytotoxicity and proliferation of nucleus pulposus **(**NP) cells. **(A)** The chemical structure of DHE. **(B)** The cell counting kit-8 **(**CCK-8) assay measured effects of the indicated concentrations of DHE on the toxicity of NP cells at 24 h. The 96-well plates contained 8,000 NP cells per well. Data are presented as the mean ± SEM. *n* = 3. ****p < 0.0001 relative to the control group. **(C–F)** Effects of the indicated concentrations of DHE on the proliferation of NP cells at 8, 24, 48, and 72 h, as measured by the CCK-8 assay. The 96-well plates contained 2,000 NP cells per well. Data are presented as the mean ± SEM. *n* = 3. **p < 0.01, ****p < 0.0001 relative to the control group.

The chemical structure of DHE is shown in Figure 1A. The CCK-8 assay was performed to determine the effects of DHE on cytotoxicity and proliferation of NP cells. For cytotoxicity analysis, 8,000 NP cells per well were seeded in 96-well plates and cultured for 24 h with complete DMEM in the presence of 0, 2.5, 5, 10, 20, or 40 μM of DHE. The cytotoxicity of DHE was calculated according to the 0 μM DHE group. DHE showed cytotoxic effects on NP cells at concentrations of 20 μM or more (Figure 1B). For proliferation analysis, 2,000 NP cells per well were seeded in 96-well plates and cultured with complete DMEM in the presence of 0, 2.5, 5, 10, 20, or 40 μM of DHE. To measure the proliferation effects of DHE, a CCK-8 assay was performed after NP cells were seeded at 8, 24, 48, and 72 h. DHE showed proliferation inhibition on NP cells at concentrations of 10 μM or more. The number of NP cells with a concentration of 2.5 μM was significantly higher than that of the 5 μM concentration at 24 and 48 h, and the difference was statistically significant. (Figures 1C–F). According to these results, 2.5 μM of DHE was used in subsequent experiments.

### DHE Alleviated TNF-α–Induced ECM Degradation and Ameliorated TNF-α–Induced Senescence of NP Cells

**FIGURE 2:**
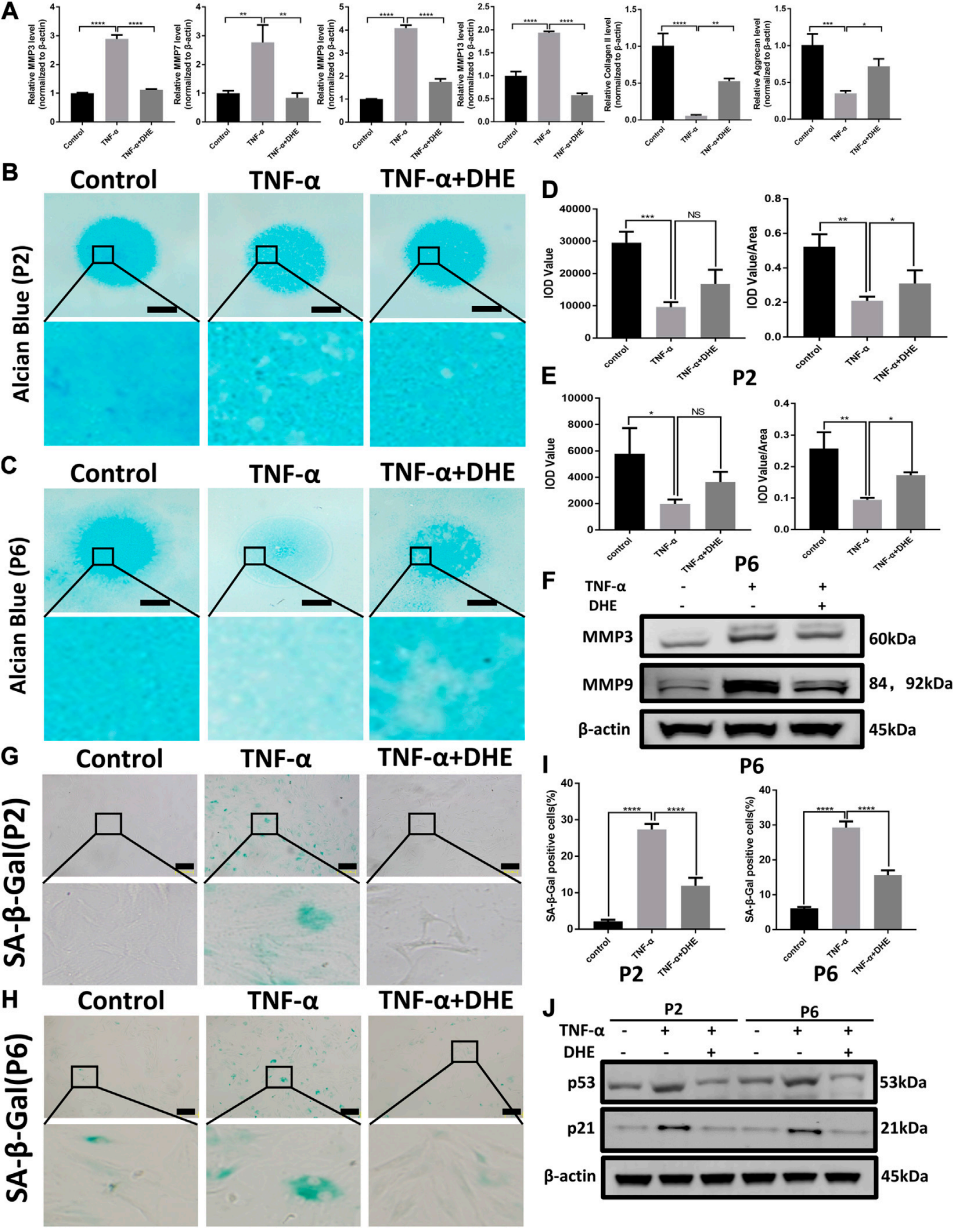
Dehydrocostus lactone (DHE) alleviated tumor necrosis factor-α (TNF-α)–induced extracellular matrix (ECM) degradation and ameliorated TNF-α–induced senescence of nucleus pulposus (NP) cells **(A)** The quantitative real-time polymerase chain reaction (RT-qPCR) was performed to detect the expression of the anabolism and catabolism genes containing matrix metalloproteins (MMPs) 3, 7, 9, and 13; collagen II; and aggrecan after treatment with TNF-α (20 ng/ml) with or without DHE (2.5 *μ*M) for 24 h. The expression of these genes was normalized to the expression of *β*-actin. *n* = 3. Data are presented as the mean ± SEM of three independent experiments. *p < 0.05, **p < 0.01, ***p < 0.001 and ****p < 0.0001 relative to the TNF-α–induced group **(B,C)** Alcian blue showed the staining of NP primary cells (P2 generation; B) and (P6 generation; C) on high-density culture after treatment with TNF-α (20 ng/ml) or TNF-α (20 ng/ml) and DHE (2.5 μM) for 9 days. Scale bar, 2 mm **(D,E)** The integrated optical density (IOD) value and IOD value/area calculations were performed to evaluate the extracellular matrix (ECM) of NP primary cells (P2 and P6) after the indicated treatment. Data are presented as the mean ± SEM of three independent experiments. *p < 0.05, **p < 0.01, and ***p < 0.001 relative to the TNF-α–induced group **(F)** Western blots demonstrated the effects of DHE on MMP3 and MMP9 expression induced by TNF-α **(G,H)** SA-β-Gal staining showed the senescence of NP primary cells (P2 generation; G) and (P6 generation; H) after indicated treatment for 3 days. Scale bar, 10 μm **(I)** The statistics of SA-β-Gal–positive NP primary cells shown in Figures 2G,H. Data are presented as the mean ± SEM of three independent experiments. ****p < 0.0001 relative to the TNF-α–induced group. **(J)** Western blots demonstrated the mitigating effects of DHE on the expression of the senescence indicators p21 and p53 induced by TNF-α in P2 and P6 generations of NP primary cells.

RT-qPCR was performed to detect the anabolism and catabolism factors of ECM. The results showed the upregulation of MMP3, MMP7, MMP9, and MMP13, which related to catabolism increasing after treatment with TNF-α. In addition, the mRNA levels of type II collagen and aggrecan (considered the main components of the ECM) decreased after stimulation by TNF-α. Those changes induced by TNF-α were partly reversed by DHE (Figure 2A). Alcian blue staining was used to detect the effects of DHE on the loss of ECM induced by TNF-α in primary NP cells. Firstly, there was no difference in alcian blue staining in the DHE alone group compared with control group (Supplementary Figures 1A,C). As shown in Figures 2B,C, the loss of ECM in the high-density culture of NP cells in the P2 and P6 generations was induced by TNF-α. DHE treatment reversed the loss of ECM under indicated concentrations (Figures 2D,E). Furthermore, western blot showed that the increase in protein production in MMP3 and MMP9 that was induced by TNF-α could be partially mitigated by DHE (Figure 2F and Supplementary Figure 2A). SA-β-Gal was used to detect the effects of DHE on the senescence induced by TNF-α in primary NP cells. And there was no difference in SA-*β*-Gal positive NP cells in the DHE alone group compared with control group (Supplementary Figures 1B,C). As shown in Figures 2G,H senescence of NP cells was induced by TNF-α in P2 and P6 NP cells. DHE treatment reversed this senescence phenomenon under the indicated concentration. The TNF-α–induced response in SA-β-Gal–positive NP cells were reduced after treatment with DHE (Figure 2I). Moreover, western blot analysis was used to detect the effect of DHE on p21 and p53 production at the protein level—production closely related to cell senescence—and showed the upregulation of these indicators (Figure 2J and Supplementary Figure 2B). Together, results show that DHE alleviates TNF-α–induced ECM degradation and the senescence of NP cells.

### Effects of DHE on TNF-α–Induced NF-κB and MAPK and dsDNA Release–Induced STING-TBK1 Activation in NP Cells

**FIGURE 3:**
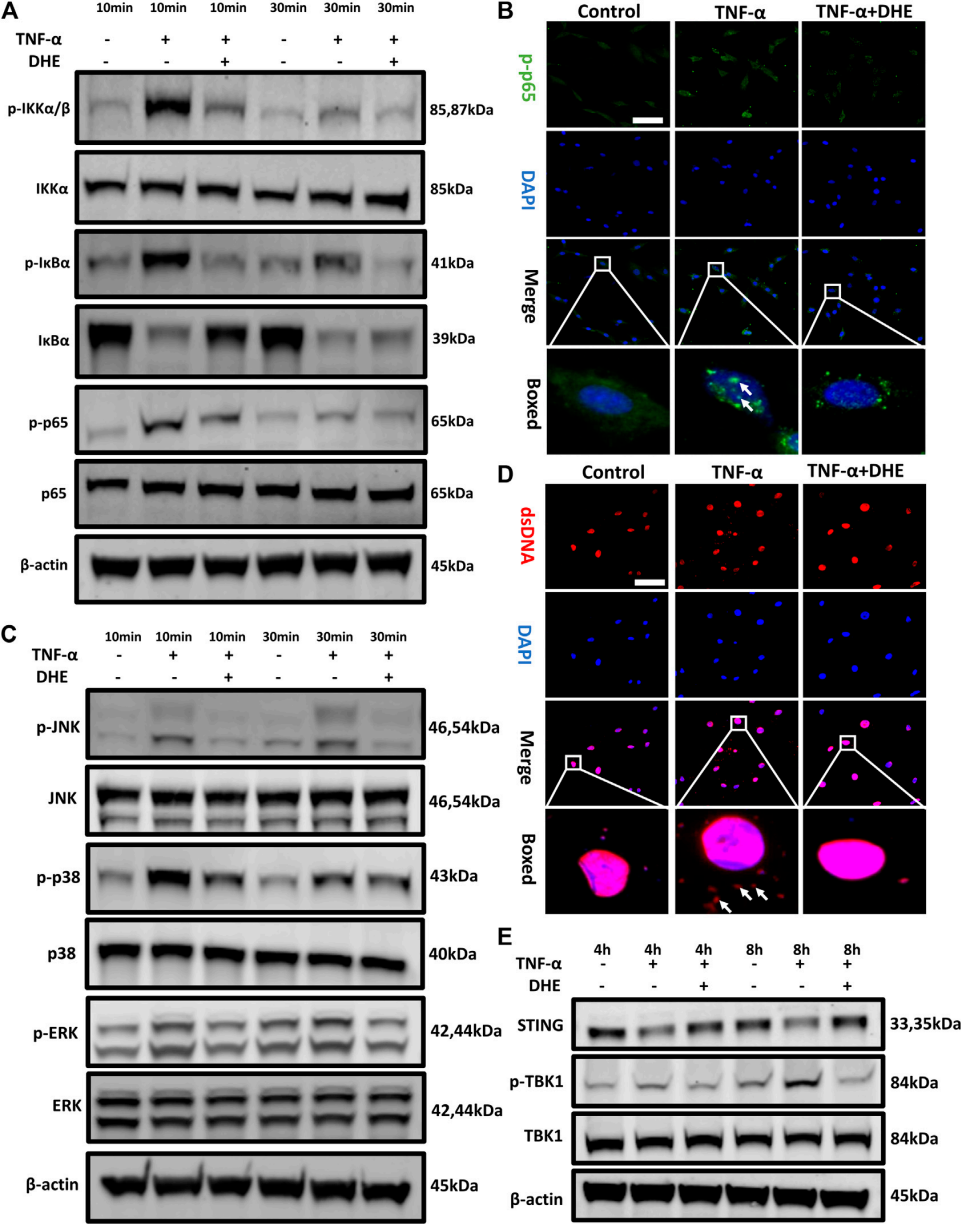
Effects of dehydrocostus lactone (DHE) on tumor necrosis factor-α (TNF-α)–induced NF-κB and MAPK as well as dsDNA release–induced STING-TBK1 activation in nucleus pulposus (NP) cells **(A)** Immunoblotting showed the DHE alleviation effects on the phosphorylation of IKKα, IκBα, and p65 and on the degeneration of total IκBα induced by TNF-α. All NP cells were pretreated with DHE (2.5 μM) for 2 h and then stimulated with TNF-α for 10 or 30 min **(B)** The phosphorylation of NF-κB p65 and nuclear translocation were detected by immunofluorescence. The arrows indicate the entry of phosphorylated p65 into the nucleus. Scale bar, 10 μm **(C)** Western blot showed the DHE alleviation effects on the phosphorylation of p38, JNK, and ERK induced by TNF-α. All NP cells were pretreated with DHE (2.5 μM) for 2 h and then stimulated with TNF-α for 10 or 30 min **(D)** The dsDNA in the cytoplasm was detected by immunofluorescence after the treatment of TNF-α (20 ng/ml) with or without DHE (2.5 μM) for 8 h. The arrows show an increase in dsDNA in the cytoplasm. Scale bar, 10 μm **(E)** Western blot showed the alleviation effects of DHE on the phosphorylation of TBK1 induced by dsDNA release and caused by TNF-α. All NP cells were treated with TNF-α (20 ng/ml) with or without DHE (2.5 μM) for 4 h or 8 h.

To investigate the mechanism underlying the effects on anabolism and catabolism of DHE, NP cells were pretreated with DHE for 2 h and then stimulated with TNF-α for another 10 or 30 min. As a result of stimulation for either duration, phosphorylation of IKKα, IκBα, and p65 was upregulated. However, these phosphorylations could be inhibited by DHE (Figure 3A and Supplementary Figure 3A). In addition, IκBα degradation induced by TNF-α was inhibited by pretreatment with DHE (Figure 3A). Immunofluorescence staining showed that DHE suppressed p65 phosphorylation and translocation from the cytosol to the nucleus (Figure 3B).

TNF-α can activate another inflammatory pathway, that of MAPK signaling. Our western blot results showed that the phosphorylation of JNK, p38, and extracellular signal-regulated kinases (ERKs) was upregulated after 10 or 30 min of TNF-α stimulation. Interestingly, phosphorylation of MAPK signaling was also inhibited by DHE (Figure 3C and Supplementary Figure 3B). To investigate the mechanism underlying the senescence of NP cells even more, we performed immunofluorescence studies on dsDNA in the cytoplasm of NP cells, which showed that dsDNA increased after TNF-α–induced senescence (Figure 3D). Finally, immunoblotting showed the decreased expression level of STING and the increased phosphorylation of TBK1 after treatment with TNF-α for 4 or 8 h. This phenomenon was ameliorated by treatment with DHE (Figure 3E and Supplementary Figure 4A). These findings suggest that the activation of the NF-κB and MAPK pathways and the cellular senescence–related dsDNA-STING signaling that is induced by TNF-α could be inhibited by DHE.

### DHE Ameliorated the Progression of IDD in a Mouse Model of Spinal Instability *in vivo*

**FIGURE 4:**
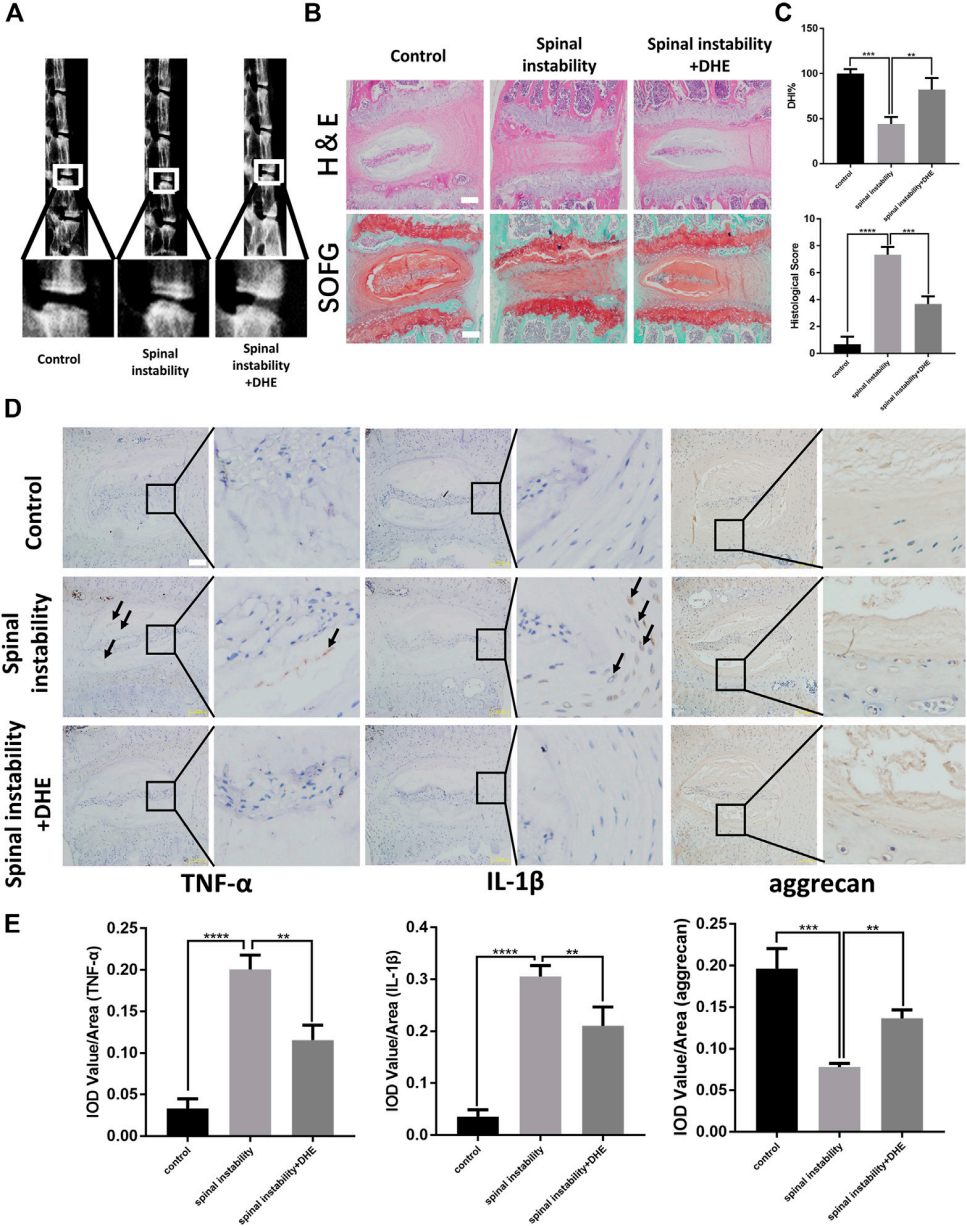
Dehydrocostus lactone (DHE) ameliorated the progression of intervertebral disc degeneration (IDD) in a spinal instability–induced mouse model *in vivo* **(A)** The lumbar spine X-ray of mice at 8 weeks after surgery from each group and a spinal instability model at 12 weeks old are shown **(B)** Hematoxylin and eosin staining and safranin O-Fast green staining of intervertebral discs (IVDs) from each group at 8 weeks after surgery. Scale bar, 10 μm **(C)** Histological scores of IVDs and quantitative analysis of disc height index (DHI, %) in mice at 8 weeks after surgery in the three groups. Scale bar, 5 μm **(D,E)** Immunohistochemical staining of interleukin (IL)-1β, TNF-α, and aggrecan expression **(D)** and their IOD Value/Area statistics **(E)** in IVDs at 8 weeks after surgery in the six groups. The arrows indicate IL-1β–and TNF-α–positive cells. Scale bar, 5 μm. Data are presented as the mean ± SEM. *n* = 6. **p < 0.01, ***p < 0.001, and ****p < 0.001 relative to the control group.

We investigated the protective effect of DHE on the progression of IDD *in vivo.* The spinal instability model of the lumbar spine was used to induce IDD. Lumbar spine X-rays and DHI data (measured in percentages) were obtained 8 weeks after the IDD model was established. The loss of intervertebral height increased significantly in the IDD model. However, the loss of intervertebral height was partly ameliorated in the IDD model when DHE was injected intraperitoneally (Figures 4A,C). Hematoxylin and eosin staining and safranin O-fast green staining of IVDs from each group showed that DHE treatment significantly alleviated the destruction of the disc structure compared with the discs in the PBS group (Figure 4B). At 8 weeks after surgery, histological scores of IVDs treated with DHE were lower than those treated with intraperitoneal injection of PBS (Figure 4C).

Immunohistochemical staining for TNF-α, IL-1*β*, and aggrecan was performed to examine the effect of DHE on inflammation and ECM *in vivo* (Figures 4D,E). The results showed that DHE alleviated the expression of TNF-α, IL-1*β*, and the degradation of aggrecan in the lumbar disc tissue of mice, which was consistent with the *in vitro* experimental results. Together, these results demonstrate that DHE is a potential therapeutic intervention for IDD.

## Discussion

This study provides evidence for the therapeutic effects of DHE in the progression of IDD. We showed that the DHE concentration of 2.5 μM had a slight effect of promoting proliferation. The IVD has a gel-like NP tissue in the center, which maintains the disc height and allows the body to bend and relieve axial mechanical stress (Roberts et al., 2006). The cellular senescence and the increased inflammatory cytokines in NP tissue that occur during IDD progression result in the obstruction of balanced ECM metabolism. This process accelerates the dysfunction of NP cells and ultimately leads to the loss of water components, type II collagen, and proteoglycans in NP tissue (Feng et al., 2016; Liu et al., 2018). IDD, with its degradation of ECM, usually precedes other spinal disorders, including disc herniation, spondylosis, and lumbar spinal stenosis. Thus, effective treatment for IDD must be explored more.

Growing research and evidence show that cellular senescence accumulates as the number of functional NP cells in IVD decreases. These changes are associated with an increased inflammatory response (Kang et al., 2015), reduced proliferation, disturbed NP tissue self-repair, and enhanced catabolism in the pathogenesis of IDD. Senescent NP cells can be highly metabolically active, with increased secretion of inflammatory cytokines and MMPs (Horn and Triantafyllopoulou 2018). This phenomenon is known as the senescent-associated secretory phenotype (Loo et al., 2020). An increasing number of studies report that inflammatory cytokines can upregulate the MMPs and break the balance between anabolism and catabolism, which results in the loss of the ECM. Senescence of NP cells and the increase in inflammatory cytokines cause a vicious cycle of degeneration and accelerate the development of IDD (Wang G. et al., 2018).

DHE is a natural sesquiterpene lactone that is isolated from the rhizome of Saussurea lappa Clarke, and it has anti-inflammatory properties. Our studies demonstrated that DHE can ameliorate TNF-α–induced inflammatory response via the NF-κB and MAPK signaling pathways and inhibit TNF-α–induced cellular senescence through the STING pathway. In addition, our *in vivo* data demonstrated that DHE effectively attenuates the progression of IDD. TNF-α is an important inflammatory factor in the activation of the NF-κB and MAPK signaling pathways (Rubin and Shapiro, 2014; Wang C. et al., 2017). Increasingly, studies have reported that TNF-α is closely related to the degeneration of IVD tissues and results in ECM degradation through activation of MMPs and inhibition of proteoglycan and collagen (Wang H. et al., 2018; Wang et al., 2020). Thus, blocking the signaling pathway induced by TNF-α could have protective effects on discs to prevent degeneration.

High-density culture demonstrated the protective effect of DHE, which reversed the degradation of ECM induced by TNF-α. DHE had a protective effect at both transcriptional and protein levels. These data suggest that DHE exhibits therapeutic effects to alleviate ECM degradation. The NF-κB and MAPK pathways have reportedly played important roles in the process of IDD (Wu et al., 2018). Notably, recent studies have linked the role of STING-TBK1 signaling with that of the NF-κB and MAPK pathways (Li and Chen, 2018; Balka et al., 2020). The relationship between these signaling pathways form a vicious circle, which accelerates the progression of IDD. The activation of NF-κB, including the phosphorylation of IKKα, IκBα, and p65 in accordance with the degradation of IκBα, is triggered by TNF-α. Finally, the downstream reaction, including catabolic enzymes and inflammatory cytokines, is upregulated. A previous study reported that DHE significantly inhibited RANKL-induced osteoclast formation via the NF-κB pathway (Hu et al., 2019). Our study demonstrated similar results—that DHE significantly inhibited the phosphorylation of IKKα, IκBα, and p65 and blocked the degradation of IκBα in TNF-α–induced NP cells. Furthermore, the activation of MAPK, including the phosphorylation of ERK, JNK, and p38, also regulates inflammatory responses. Increasingly, studies have reported that MAPK signaling is closely related to matrix synthesis and ECM degradation in the progression of IDD (Rubin and Shapiro, 2014; Ge et al., 2020). Surprisingly, our study demonstrated downregulated phosphorylation effects of JNK, ERK, and p38.

**FIGURE 5:**
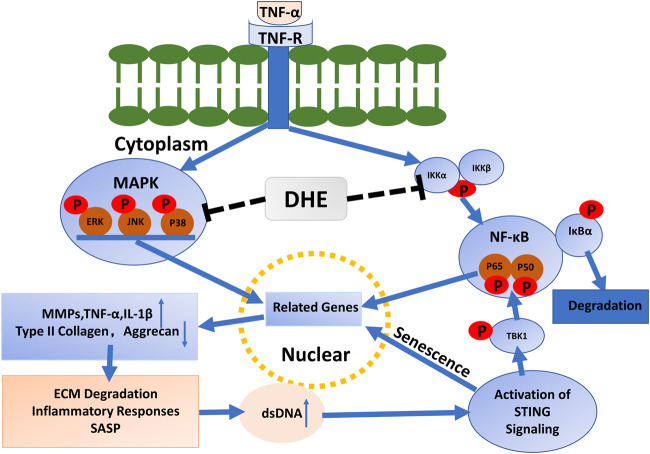
Mechanisms driving the effects of dehydrocostus lactone (DHE) in tumor necrosis factor-α (TNF-α)–induced cellular senescence and inflammatory responses in nucleus pulposus (NP) cells.

In this study, we investigated the anti-senescence effects of DHE in TNF-α–activated NP cells. We observed that, after activation with TNF-α, the P2 and P6 generations of NP cells had increased SA-β-Gal–positive cells. This phenomenon was inhibited after addition of DHE. Immunofluorescence demonstrated that dsDNA in the cytoplasm increased after stimulation with TNF-α and decreased after the use of DHE together with TNF-α. Mechanistically, the dsDNA activated the STING pathway and caused the senescence of NP cells. DHE inhibited the increase of dsDNA in the cytoplasm of NP cells, thus relieving the activation of STING pathway and ultimately ameliorating the senescence of NP cells. Interestingly, we observed STING degradation after TNF-α stimulation of NP cells; this phenomenon is in accordance with a previous study (Konno et al., 2013). Previous studies have also shown that phosphorylation of TBK1 further activates the NF-κB pathway (Reilly et al., 2013; Balka et al., 2020). These results confirm our previous speculation that STING-TBK1/NF-κB and MAPK signaling create a vicious cycle of inflammatory cascades that accelerate IDD (Figure 5).

Considered together, the results of this study show that DHE can ameliorate the senescence of NP cells via reduced STING signaling activation and by alleviating inflammatory factors through inhibition of NF-κB and MAPK pathways. Moreover, our *in vivo* data show the protective function of DHE in the progression of IDD. Notably, clinically available drugs containing DHE, such as compound ancklandia and berberine tablets, have been used for their anti-inflammatory actions to treat digestive tract diseases. Therefore, DHE may be considered safe and reliable for clinical use. On the basis of the beneficial effects of DHE on IDD observed in this study both *in vivo* and *in vitro*—including reductions of inflammation, ECM catabolism, and cellular senescence—DHE may be considered as a treatment for IDD, either by oral administration or as a local IVD injection.

## Data Availability

The raw data supporting the conclusions of this article will be made available by the authors, without undue reservation, to any qualified researcher.
