# Supplementary material for: Dehydrocostus Lactone Attenuates the Senescence of Nucleus Pulposus Cells and Ameliorates Intervertebral Disc Degeneration via Inhibition of STING-TBK1/NF-κB and MAPK Signaling
Source: Front Pharmacol. 2021 Apr 14;12:641098. doi: 10.3389/fphar.2021.641098 (PMC8079987; doi:10.3389/fphar.2021.641098)
Supplement: Supplementary file 1 [file datasheet1.pdf]

Supplementary Figures

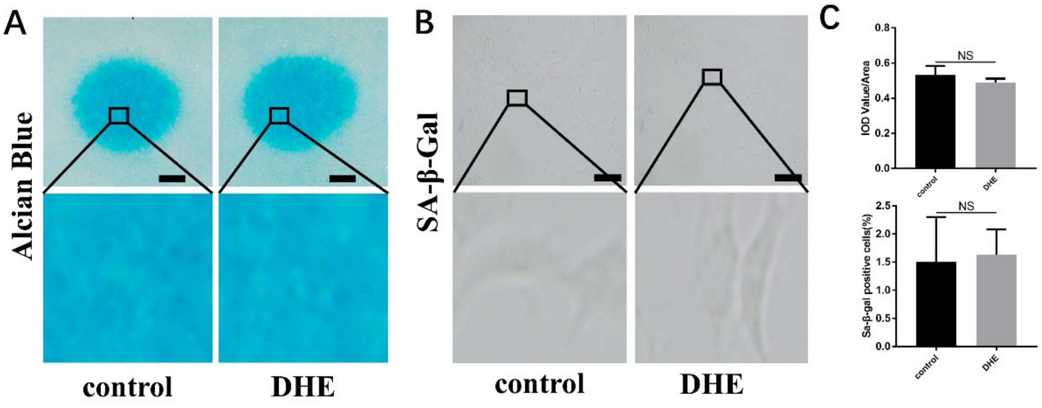

Supplementary Figure 1 | DHE alone had no impact on extracellular matrix (ECM) and senescence of NP Cells **(A)** Alcian blue showed the staining of NP primary cells (P2 generation) on high-density culture after treatment with DHE (2.5 μM) for 9 days. Scale bar, 2 mm. **(B)** Sa-β-gal staining showed the senescence of NP primary cells (P2 generation) after DHE (2.5 μM) treatment for 3 days. Scale bar, 10 μm. **(C)** The statistics of IOD value/area calculations and SA-β-Gal–positive NP primary cells in A and B. Data are presented as the mean ± SEM of three independent experiments. NS, nonsignificant.

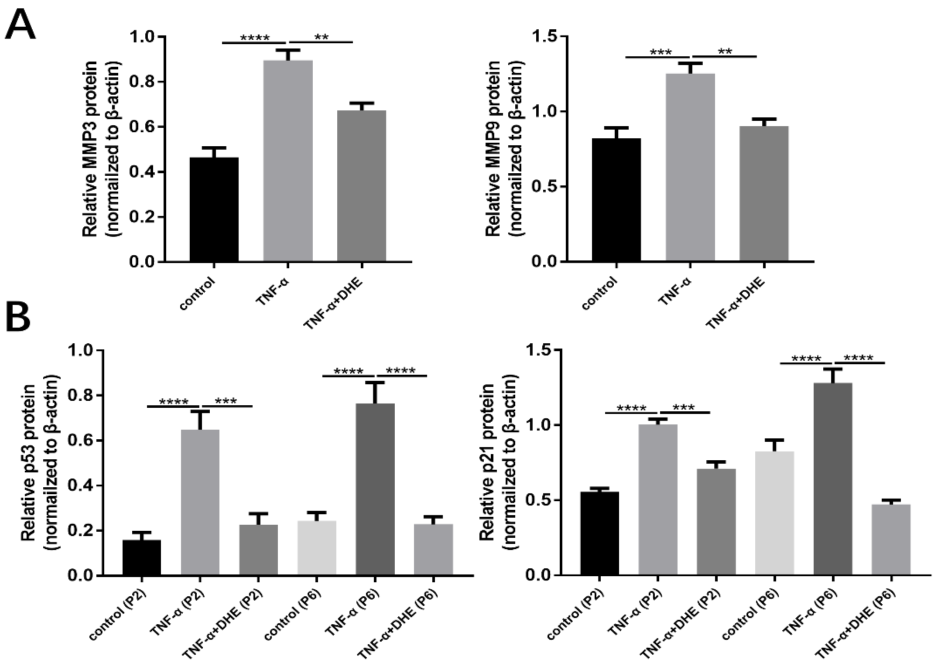

Supplementary Figure 2 | Western blotting statistics in Figure 2F and 2J. **(A)** Densitometric analysis of MMP3 and MMP9 normalized to β-actin related to Fig 2F. **(B)** Densitometric analysis of p21 and p53 normalized to β-actin related to Fig 2J. Values shown are mean ± SEM from 3 independent experiments. NS, nonsignificant; ****$p < 0.0001$, ***$p < 0.001$, **$p < 0.01$.

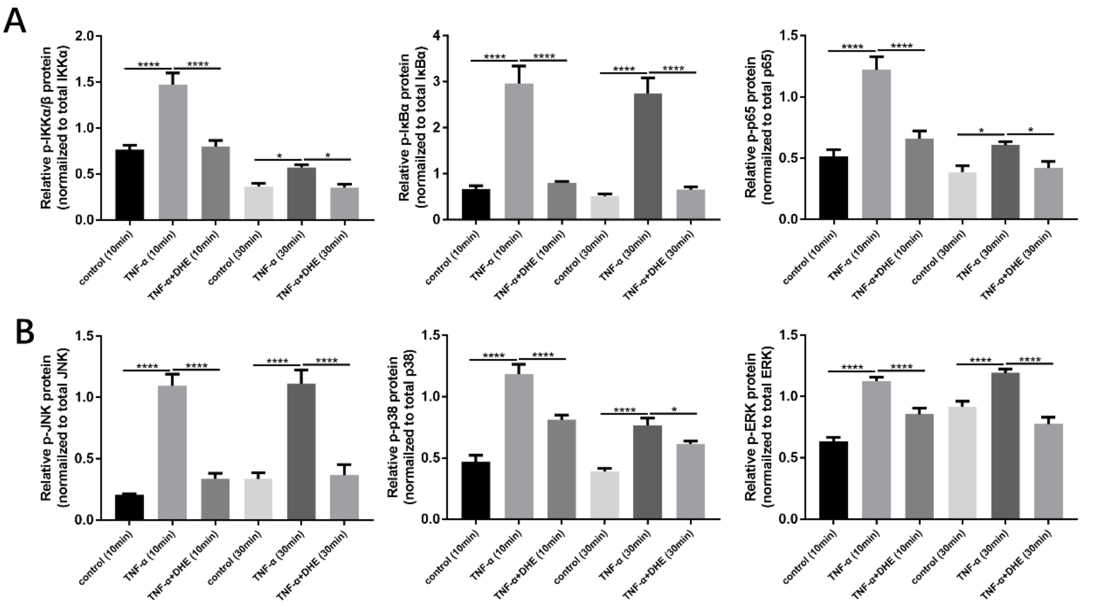

Supplementary Figure 3 | Western blotting statistics in Figure 3A and 3C. **(A)** Densitometric analysis of p-IKKα/β, p-IκBα and p-p65 normalized to total IKKα, IκBα and p65 respectively related to Fig 3A. **(B)** Densitometric analysis of p-JNK, p-p38 and p-ERK normalized to total JNK, p38 and ERK respectively related to Fig 3C. Values shown are mean ± SEM from 3 independent experiments. NS, nonsignificant; ****$p < 0.0001$, *$p < 0.05$.

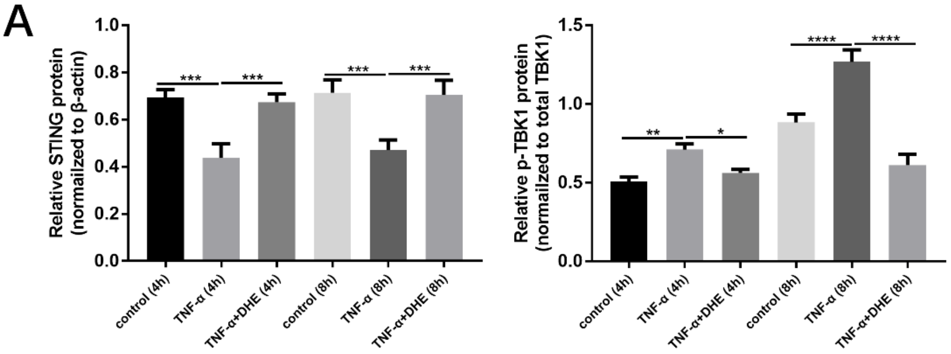

Supplementary Figure 4 | Western blotting statistics in Figure 3E. **(A)** Densitometric analysis of STING normalized to β-actin and p-TBK1 normalized to total TBK1 related to Fig 3E. Values shown are mean ± SEM from 3 independent experiments. NS, nonsignificant; ****$p < 0.0001$, ***$p < 0.001$, **$p < 0.01$, *$p < 0.05$.
