# Supplementary material for: Dehydrocostus Lactone Attenuates the Senescence of Nucleus Pulposus Cells and Ameliorates Intervertebral Disc Degeneration via Inhibition of STING-TBK1/NF-κB and MAPK Signaling
Source: Front Pharmacol. 2021 Apr 14;12:641098. doi: 10.3389/fphar.2021.641098 (PMC8079987; doi:10.3389/fphar.2021.641098)
Supplement: Supplementary file 2 [file table1.pdf]

Supplementary Table 1

PCR primers information

Gene	Accession Number	Description	5'-Primer-3'
MMP3	NM_010809.2	F<br>R	CCCTGCAACCGTGAAGAAGA<br>GACAGCATCCACCCTTGAGT
MMP7	NM_010810.5	F<br>R	CCCTGTTCTGCTTTGTGTGTCA<br>GGGGGAGAGTTTTCCAGTCA
MMP9	NM_013599.4	F<br>R	CCGACTTTTGTGGTCTTCCCC<br>ATGTCTCGCGGCAAGTCTTC
MMP13	NM_008607.2	F<br>R	AGAAGTGTGACCCAGCCCTA<br>GGTCACGGGATGGATGTTCA
Col2a1	NM_053593.2	F<br>R	GTGTGACACTGGGAATGTCCT<br>CTTGGCCCTAATTTTCCACTGGC
Aggrecan	NM_001361500.1	F<br>R	TGCAGACATTGACGAGTGCC<br>AGAGAGTGTCCGTCAGACCA
β-actin	NM_031144.3	F<br>R	ACCCGCGAGTACAACCTTC<br>ATGCCGTGTTCAATGGGGTA
